# Structuration in the Interface of Direct and Reversed Micelles of Sucrose Esters, Studied by Fluorescent Techniques

**DOI:** 10.1371/journal.pone.0123669

**Published:** 2015-04-23

**Authors:** Catalina Sandoval, Anakenna Ortega, Susana A. Sanchez, Javier Morales, German Gunther

**Affiliations:** 1 Departamento de Química Orgánica y Fisicoquímica, Facultad de Ciencias Químicas y Farmacéuticas, Universidad de Chile, Santiago, Chile; 2 Departamento de Polímeros, Facultad de Química, Universidad de Concepción, Concepción, Chile; 3 Departamento de Ciencias y Tecnología Farmacéuticas, Facultad de Ciencias Químicas y Farmacéuticas, Universidad de Chile, Santiago, Chile; Brandeis University, UNITED STATES

## Abstract

**Background:**

Reactors found in nature can be described as micro-heterogeneous systems, where media involved in each micro-environment can behave in a markedly different way compared with the properties of the bulk solution. The presence of water molecules in micro-organized assemblies is of paramount importance for many chemical processes, ranging from biology to environmental science. Self-organized molecular assembled systems are frequently used to study dynamics of water molecules because are the simplest models mimicking biological membranes. The hydrogen bonds between sucrose and water molecules are described to be stronger (or more extensive) than the ones between water molecules themselves. In this work, we studied the capability of sucrose moiety, attached to alkyl chains of different length, as a surface blocking agent at the water-interface and we compared its properties with those of polyethylenglycol, a well-known agent used for this purposes. Published studies in this topic mainly refer to the micellization process and the stability of mixed surfactant systems using glycosides. We are interested in the effect induced by the presence of sucrose monoesters at the interface (direct and reverse micelles) and at the palisade (mixtures with Triton X-100). We believe that the different functional group (ester), the position of alkyl chain (6-O) and the huge capability of sucrose to interact with water will dramatically change the water structuration at the interface and at the palisade, generating new possibilities for technological applications of these systems.

**Results:**

Our time resolved and steady state fluorescence experiments in pure SEs micelles show that sucrose moieties are able to interact with a high number of water molecules promoting water structuration and increased viscosity. These results also indicate that the barrier formed by sucrose moieties on the surface of pure micelles is more effective than the polyoxyethylene palisade of Triton X-100. The fluorescence quenching experiments of SEs at the palisade of Triton X-100 micelles indicate a blocking effect dependent on the number of methylene units present in the hydrophobic tail of the surfactant. A remarkable blocking effect is observed when there is a match in size between the hydrophobic regions forming the apolar core (lauryl SE/ Triton X-100). This blocking effect disappears when a mismatch in size between hydrophobic tails, exists due to the disturbing effect on the micelle core.

## Introduction

Reactors found in nature can be described as micro-heterogeneous systems, where media involved in each micro-environment can behave in a markedly different way compared with the properties of the bulk solution. For many chemical reactions properties such as rates, mechanisms and even regio- and stereochemistry are different if they are measured in the micellar media or in the pure bulk solvents. The presence of water molecules in micro-organized assemblies is of paramount importance for many chemical processes, ranging from biology to environmental science. The structuration of water molecules is heavily influenced by the chemical environment, due to hydrogen bonds, interactions with ions, and confinement on the nanometer scale.[1] Surfactant self-organized molecular assembled systems are the simplest models mimicking biological membranes, and they are frequently used to study the dynamics of water molecules.

Water shows very different properties depending if the molecules are confined with restricted movement (with important degree of structuration), or they are in bulk solution. There are several examples of these observations in micellar systems in the literature.[2,3] For example, Borsarelli et al. studied the intramolecular photooxidation of ruthenium cyano-complexes (Ru(bpy)(CN)_4_^2-^) by LIOAS in AOT reverse micelles. They demonstrated that at high R values (water to surfactant ratio), parameters such as the structural volume change, the emission quantum yields and the lifetimes related with the MLCT (metal to ligand charge transfer state) formation showed similar values to the ones determined in homogeneous water solution They also showed that at low R values, the rigidity of the water core is reflected in a much smaller expansion and somewhat higher emission lifetime and quantum yield.[2]

Micellar polarity (dielectric constant) plays an important role in incorporation (solubilizing capacity) of drugs into micelles. The actual knowledge indicates that the water molecules responsible for the observed solvation process in several systems[4] are the ones confined around the probe at the micelar palisade layer. Kumbhakar et Al. studied Triton micelles, and showed a clear correlation between solvation dynamics and changes in the micellar structure, size and degree of hydration.[5] The palisade layer in Triton micelles has been described by several authors, and is postulated to be mainly composed by the oxyethylene groups of the surfactant molecules and a large number of water molecules hydrogen bonded, either to the oxyethylene units (referred as bound water) or among themselves (called free water).[5,6] Consequently and depending on the polarity of the choosen probe, its location will be superficial or deeply inside in the palisade layer. For instance, apolar probes will locate inside the micellar hydrophobic core entangled with the surfactant aliphatic chains. Fluorescence anisotropy measurements[7] indicate that microviscosity inside the palisade layer of Triton micelles is inversely dependent on the number of oxyethylene units present, for example, the structure of the palisade layer of TX-165 micelles would be quite loose and with a high degree of hydration when compared to TX-100 micelle palisade.

The hydrogen bonds between sucrose and water molecules are described to be stronger (or more extensive) than the ones between water molecules themselves.[8,9] Besides, intramolecular hydrogen bonds may reduce the number of possible hydrogen bonds with the solvent molecules increasing the rigidity of the sugar moiety.[9] In this work, we studied the capabilities of sucrose moiety as a surface blocking agent and we compared them with those of polyethylenglycol, a well-stablished agent used for this purposes.[10,11] We used these sugar-based nonionic surfactants (sucrose esters) due to their interesting physical chemical properties, their biodegradability and the fact that their headgroups can be obtained from renewable resources.[12] This kind of surface active compounds have an important number of applications in productive processes, but there is no much information on the effect of the sugar structure (polyol) on these applications. Several studies exist on the characterization of their self-assembling properties,[13,14] their modifying / solubilizing effect on lipid membranes,[15–17] and their role in membrane enzymes extraction.[18] Moreover, these non-ionic surfactants play a role in the solubilization of drugs, being a potential replacement to the widely used glycerides in the formulation of lipid-based drug delivery systems.[14,19] They have been shown to stabilize protein-loaded micro-particles.[20] and have been employed in the design of vaccines.[21]

In this work we studied the structuration of water molecules in micelles and reversed micelles, taking advantage of the capability of 6-O-sucrose esters to form both types of association aggregates. We also examined the interactions in mixtures of surfactants. These mixtures often show synergistic interactions, which could result in an improvement of specific properties. Among these systems, those consisting of ionic and non-ionic species are the most studied.[22–26] In contrast, mixtures of non-ionic surfactants, which characterized by the absence of strong interactions, [22] have not received much attention. There are few reports on mixtures of hexaoxyethylene dodecyl ether with glycosides and they show low degree of interactions and, therefore, almost ideal behavior.[22,27,28] In this work, we studied mixtures of Triton X-100 with different proportions of sucrose monoesters derived from decyl, lauryl and palmityl acids. Existing studies in the literature are mainly related with the micellization process and the stability of mixed surfactant systems involving glycosides, we are interested in the study of the effect in the interface (direct and reverse micelles) and at the palisade (mixtures with Triton X-100) caused by the presence of different sucrose monoesters. We believe that the different chemical functionality involved (ester), the position of alkyl chain (6-O) and the huge capacity of sucrose to interact with water will generate new and interesting results rising the number application related properties available. Since micellar polarity probably plays a role in the solubilizing capacity of drugs, we hypothesize that the confined water molecules in the micellar palisade layer (responsible for the solvation process in the present systems),[4] would be perturbed or altered by the presence of increasing amounts of sucrose.

## Experimental Section

### Materials

Sucrose monoesters, β-d-fructofuranosyl-6-O-decyl-α-d-glucopyranoside (MCS, Mono capryl sucrose, C10), β-d-fructofuranosyl-6-O-lauryl-α-d-glucopyranoside (MLS, Mono lauryl sucrose, C12), β-d-fructofuranosyl-6-O-palmityl-α-d-glucopyranoside (MPS, Mono palmityl sucrose, C16) and β-d-fructofuranosyl-6-O-steraryl-α-d-glucopyranoside (MSS, Mono stearyl sucrose, C16) were synthesized in our laboratory by a modification of the Vlahov method,[29] the relation between sucrose complex and acyl chloride was changed to two to one. The reaction yields a mixture of monoesters (mainly 6-*O* and presumably a small quantity of 1-*O*), accompanied with low amounts of di- and tri-esters. Chromatography on silica column was employed to isolate pure 6-O monoesters of all synthesized compounds (briefly, the pre-purified reaction mixture was solubilized in chloroform and eluted from a semi-preparative silica gel column by using a stepped gradient of chloroform: methanol (15:1 to 4:1) as mobile phase. Thin layer chromatography (using chloroform: methanol 4:1 as mobile phase and staining with a butanolic solution of urea–orthophosphoric acid) showed one compound in the purified sample. The NMR spectra, obtained in a Bruker ADX 300 spectrometer, with DMSO*d*-6 containing 5% of CD_3_OD to avoid micellization, agree with previously reported spectra for monoesters.[29,30]

6-Dodecanoyl-2-dimethylaminonaphthalene (Laurdan), from Molecular Probes and dodecyl pyridinium chloride (DPC), Bis(2-ethylhexyl) sulfosuccinate sodium salt (AOT), Rose Bengal (RB), 8-hydroxypyrene-1,3,6-trisulfonic acid (Pyranine) and Triton X-100 from Sigma were employed as received. Pyrene, from Merck, was recrystallized twice from ethanol. Solvents from Merck were all HPLC quality. Water was purified with Milli-Q equipment from Waters.

**Fig 1:**
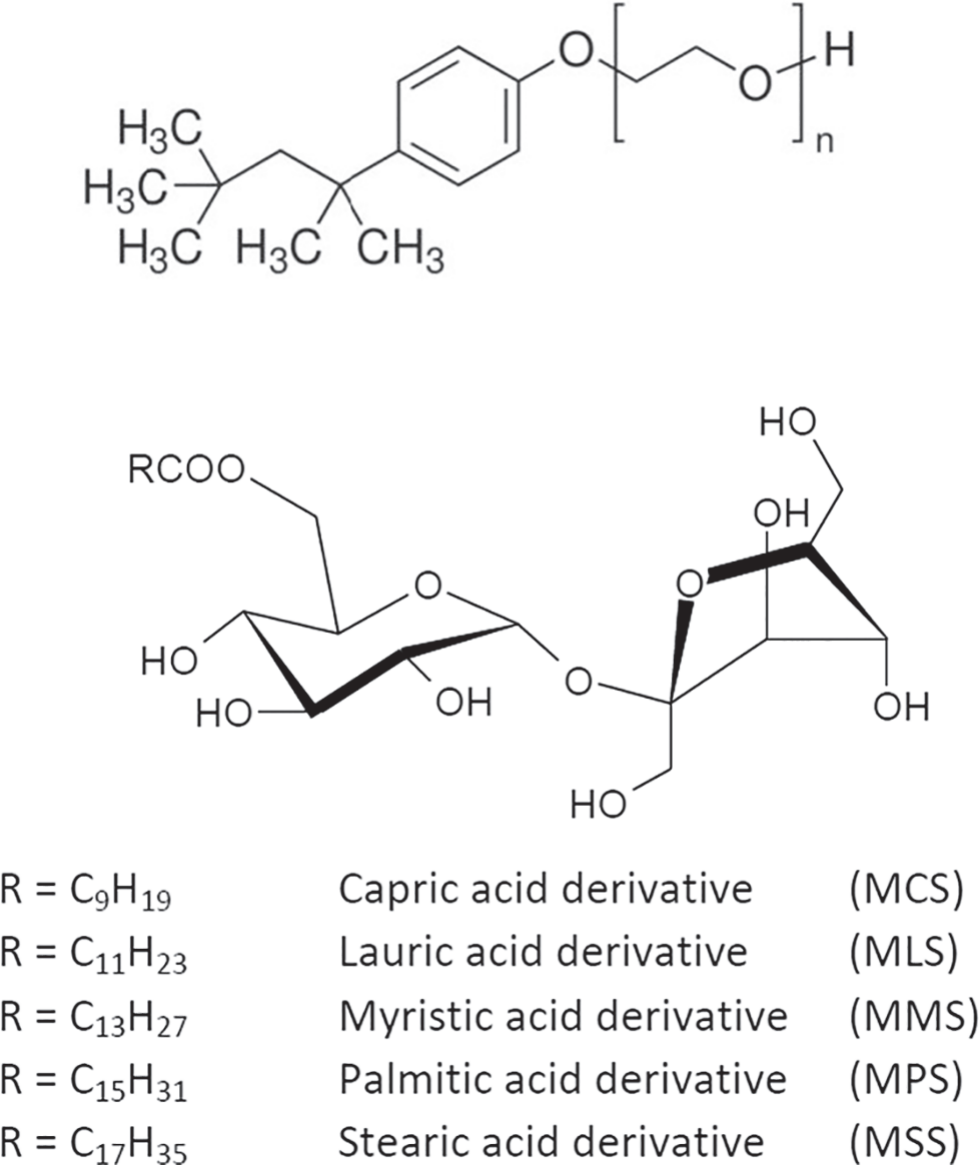
Chemical structure of Triton X-100 and Sucrose esters.

Chemical structures of the studied compounds are summarized in Fig 1.

## Methods

### 1. Steady-state fluorescence measurements

All fluorescence emission measurements were performed in a Fluorolog τ-2 spectrofluorimeter (formerly Spex, now Horiba) thermostated at 25°C, emission and excitation slits were kept at 0.5 mm.

#### Reversed micelles aqueous pool characterization

**Fig 2:**
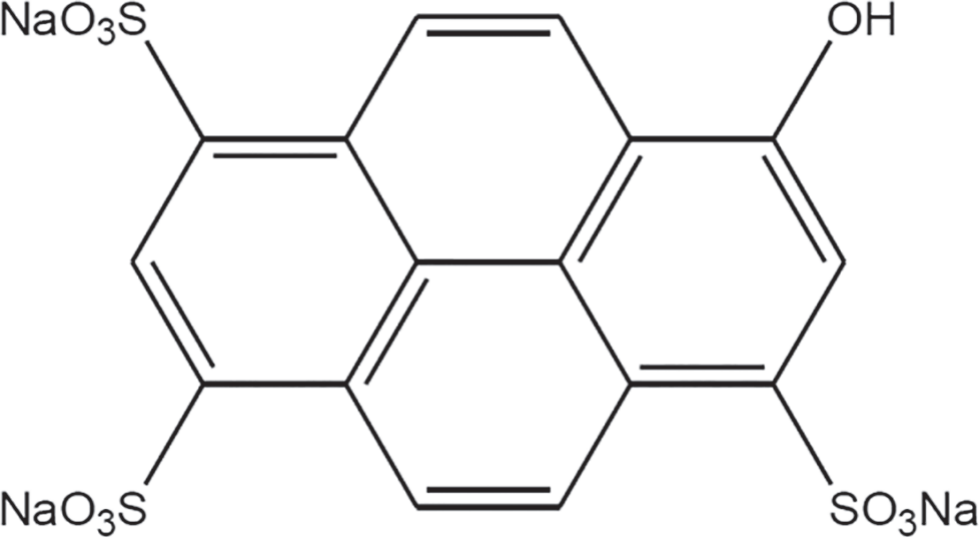
Chemical structure of sodium 8-hydroxy-1,3,6-pyrenetrisulfonate, Pyranine.

In order to study the structuration of water inside the aqueous pool of reversed micelles, we used Pyranine (8-hydroxypyrene-1,3,6-trisulfonic acid). This fluorescent compound is usually employed as pH probe, and its chemical structure is shown in Fig 2. It is a water-soluble fluorophore derived from Pyrene, whose fluorescent excitation and emission spectra are highly dependent on pH, and it is commonly employed in biochemical and biophysical research, since the pH changes can be easily quantified using a pH scale.[31]

The pK_a_ of this probe is reported to be 7.22, but this value has been informed to be dependent on the composition of the medium.[31] To determine pH by using Pyranine as probe, the procedure consists on measuring the ratio of intensity emission at 510 nm using two excitation wavelengths (415 and 460nm). Emission intensity, with excitation at 460 nm, is related to the amount of non-protonated pyranine (ionized 8-hydroxy group) and emission intensity with excitation at 415 nm (an isosbestic point of Pyranine) accounts for the total amount of Pyranine present in the system (protonated plus non-protonated). Thus, the 460/415-nm excitation ratio (with emission observation at 510nm, as mentioned) is directly related with Pyranine ionization degree, and several reported equations allow determining the pH value.[31,32]

**Fig 3:**
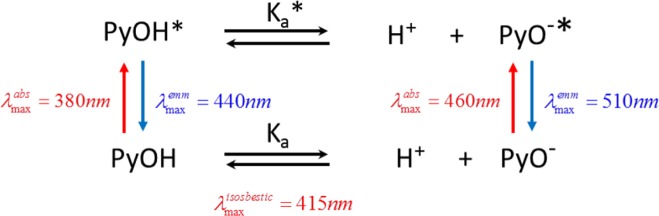
Diagrammatic scheme of the equilibria of Pyranine in both, ground and excited state. Absorption and emission maxima wavelength are also included.

In order to fully understand the photochemistry associated to the acid-base equilibrium of Pyranine Fig 3 is included. In neutral aqueous solution, Pyranine molecule in excited state suffers complete acid dissociation (reported pKa* equal to 0,4[33]) and only one emission maximum centered at 510nm is observed corresponding to the deprotonated excited form of the probe (PyO^-^*). When the probe is incorporated into reversed micelles such as AOT,[34] two emission bands can be observed, at 440nm and 510nm, corresponding to the protonated excited (PyOH*) and non-protonated excited (PyO^-^*) species, respectively. When the amount of water is increased, with the concomitant increase of micellar size, emission centered at 510nm grows and the emission at 440nm decreases, clear indication of conversion of PyOH* to PyO^-^*.

#### Micropolarity and microviscosity measurements

Micropolarity determinations were performed by employing the fluorescent probe Pyrene, and the Py scale proposed by Dong et al,[35] where the ratio of emission intensities at 372 nm and 384 nm (I/III) is related with the polarity sensed by the probe. In these measurements Pyrene concentration was kept low (below 1 μM) to avoid excimer formation.

Microviscosity determinations were performed by using the fluorescent probe Laurdan and its generalized polarization (GP) parameter proposed by Parasassi et al.[36]

$$GP=\frac{I_{440}-I_{490}}{I_{440}+I_{490}}$$ (Eq 1)

Where I_440_ and I_490_ correspond to the emission intensity at the respective wavelengths after excitation at 364nm.

### 2. Time resolved fluorescence measurements

Time resolved lifetime measurements were performed in a customized PicoQuant Fluotime 200 system. For singlet oxygen emission determinations, Rose Bengal was employed as sensitizer, excitation at 532 nm was achieved with a laser FTSS355-Q3, (Crystal Laser, Berlin, Germany) working at 1 kHz repetition rate. For the detection at 1270nm (Wavelength selection was achieved with an interference filter) a NIR PMT H10330A (Hamamatsu) was used. The luminescence Photon counting was achieved with a multichannel scaler (PicoQuant’s Nanoharp 200). Cell holder was thermostated at 25.0 ± 0.1°C.

For Pyrene quenching experiments using the ionic surfactant dodecyl pyridinium chloride (DPC) as quencher, excitation was achieved with a LED (PLS-330, Picoquant, Germany) working at 1 MHz repetition rate at 334 nm and emission was detected at 380 nm, with emission slits kept at 1.0 mm. The luminescence Photon counting was achieved with a multichannel scaler (PicoQuant’s Timeharp 200). For all experiments cell holder was thermostated at 25.0 ± 0.1°C.

Time-resolved emission signals were analyzed to extract lifetime values and kinetic information, using ORIGIN 8.6 or FLUOFIT software

#### Indirect evaluation of Rose Bengal triplet state lifetime

We studied water structuration using sensitizer triplet lifetime *τ*_*T*_. Triplet lifetime is dependent on media and water structuration has a noticeable effect on this parameter. Rose Bengal is a dye commonly used to produce singlet oxygen, O_2_(^1^Δ_g_). Singlet oxygen is an excited specie of molecular oxygen that cannot be generated by direct absorption of electromagnetic radiation, it must receive the required excitation energy from another excited molecule, usually called dye or sensitizer. The excited state lifetime of the sensitizer is dependent on the media, and water structuration has a noticeable effect on its value. The excitation process of photosensitization, involves at least two processes: photosensitizer excitation (to its singlet or triplet excited states), followed by energy transfer to molecular oxygen at ground state. If the lifetime of the first singlet excited state of the dye is long (in the order of 20ns), molecular oxygen can favor intersystem crossing to the triplet state of sensitizer (yielding also singlet oxygen). Triplet excited states, with longer lifetimes, are more prone to interact with molecular oxygen and the generation of O_2_(^1^Δ_g_) is favored. On the other side, if the lifetime of the excited singlet state is too short (in the order of nanoseconds) or the energy gap between triplet and singlet is smaller than the O_2_(^1^Δ_g_) energy, photosensitization will involve only the triplet states of the sensitizer. When this is the case, infrared emission of excited oxygen fits to the following bi-exponential equation

I(t)=A(e−tτ1−e−tτ2) (Eq 2)

The constant A is empirical and involves instrumental factors and several kinetic parameters and ground state oxygen and excited sensitizer concentrations.[37,38] Exponential terms correspond to a growing signal (related with τ_1_) and to a decay signal (related with τ_2_). Intensity is proportional to O_2_(^1^Δ_g_) concentration, and one exponential term is related with sensitizer triplet state lifetime, *τ*_*T*_, (which is a function of molecular oxygen concentration, employing Wilkinson notation: 1/τT=kdT+kdO2[O2(Σ3g)]) and the other is related with singlet oxygen lifetime, *τ*_*d*_, (which in absence of quenchers equals *τ*_Δ_).

**Fig 4:**
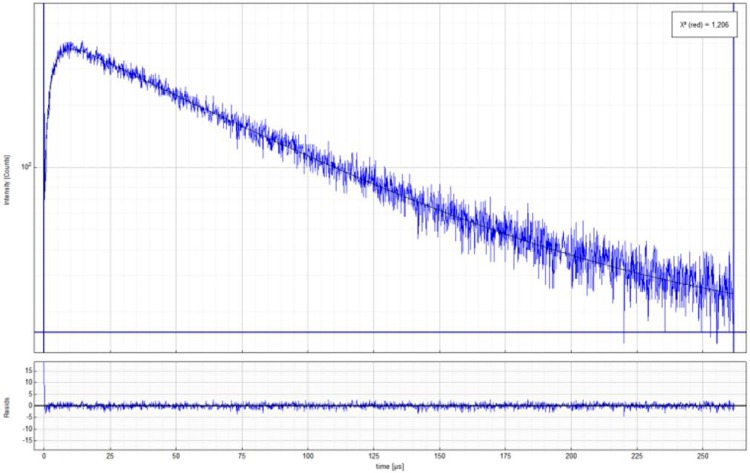
Singlet oxygen phosphorescence decay of O_2_(^1^Δ_g_) in D_2_O (air equilibrated), employing Bengal Rose as sensitizer. Signal growth corresponds to dye triplet lifetime (3,4μs) and decay is excited oxygen lifetime (66μs). Lower panel shows fitting residuals.

The relationship between *τ*_1_ and *τ*_2_ with *τ*_Δ_ and *τ*_*T*_ is not always direct, for most solvents in equilibrium with air, *τ*_*T*_ < *τ*_Δ_. In the case of Rose Bengal, the growing term of Eq 2 involves sensitizer triplet lifetime (*τ*_1_ = *τ*_*T*_) and the decay corresponds to O_2_(^1^Δ_g_) lifetime (*τ*_2_ = *τ*_Δ_). When the media is a solvent where singlet oxygen is efficiently quenched (being water the paramount case) the two lifetimes become similar, and the only way to assign the time parameters of Eq 2 is with additional flash photolysis experiments (determination of sensitizer triplet lifetime). Fig 4 shows the time dependence of O_2_(^1^Δ_g_) phosphorescence in deuterated water in equilibrium with air.

#### Decay parameters of Pyrene emission from micellar systems

The fluorescence decay for Pyrene (immersed in micellar systems) using DPC as quencher will be analyzed considering two possible scenarios: (i) the quencher molecules remain in the micelle and there is not exchange of them with other micelles and, (ii) the quencher molecules are exchanged among micelles, diffusing from one micelle to another through the water phase. When both, Pyrene and the quencher are, either strongly bound to the micelle or the diffusion of the molecule through the water phase is in the time scale of the excited state of the probe, the intensity decay for Pyrene can be described by:

$$I\left(t\right)=I_{0}e^{-k_{0}t+N\left(e^{-k_{q}t-1}\right)}$$ (Eq 3)

Where: k_o_ is the reciprocal of the Pyrene lifetime in absence of the quencher (determined in independent experiments), N is the quencher mean occupation number ([quencher]/[micelles]), and k_q_ is the pseudo-first-order rate constant for intra-micellar quenching.

A more complex kinetic scheme is needed to account for inter-micellar quencher migration, the equations involved in this case are:

$$I\left(t\right)=I_{0}e^{-A_{1}t+A_{2}\left(e^{-A_{3}t-1}\right)}$$ (Eq 4)

$$A_{1}=k_{0}+\frac{k_{q}k_{-}N}{k_{q}+k_{-}}$$ (Eq 5)

$$A_{2}=\frac{Nk_{q}^{2}}{{\left(k_{q}+k_{-}\right)}^{2}}$$ (Eq 6)

$$A_{3}=k_{q}+k_{-}$$ (Eq 7)

Where k_o_, k_q_ and N have the same meaning and k_+_ and k_-_ correspond to the entrance and exit rate constant for a quencher, related with enter and to leave the micelle.[39]

From the fitting with these models, besides the kinetic information of the quencher behavior obtained, the mean occupation number (N) allows to obtain an estimate of the mean aggregation number of the micelles.[40] Additionally kinetic data can be directly related with properties like accessibility (quencher access) or microviscosity (quencher or probe diffusion) of the site where probe and quencher are located.

## Results and Discussion

### 1 Study of water structuration in reversed micelles of sucrose esters

#### 1.1 Pyranine emission study

**Fig 5:**
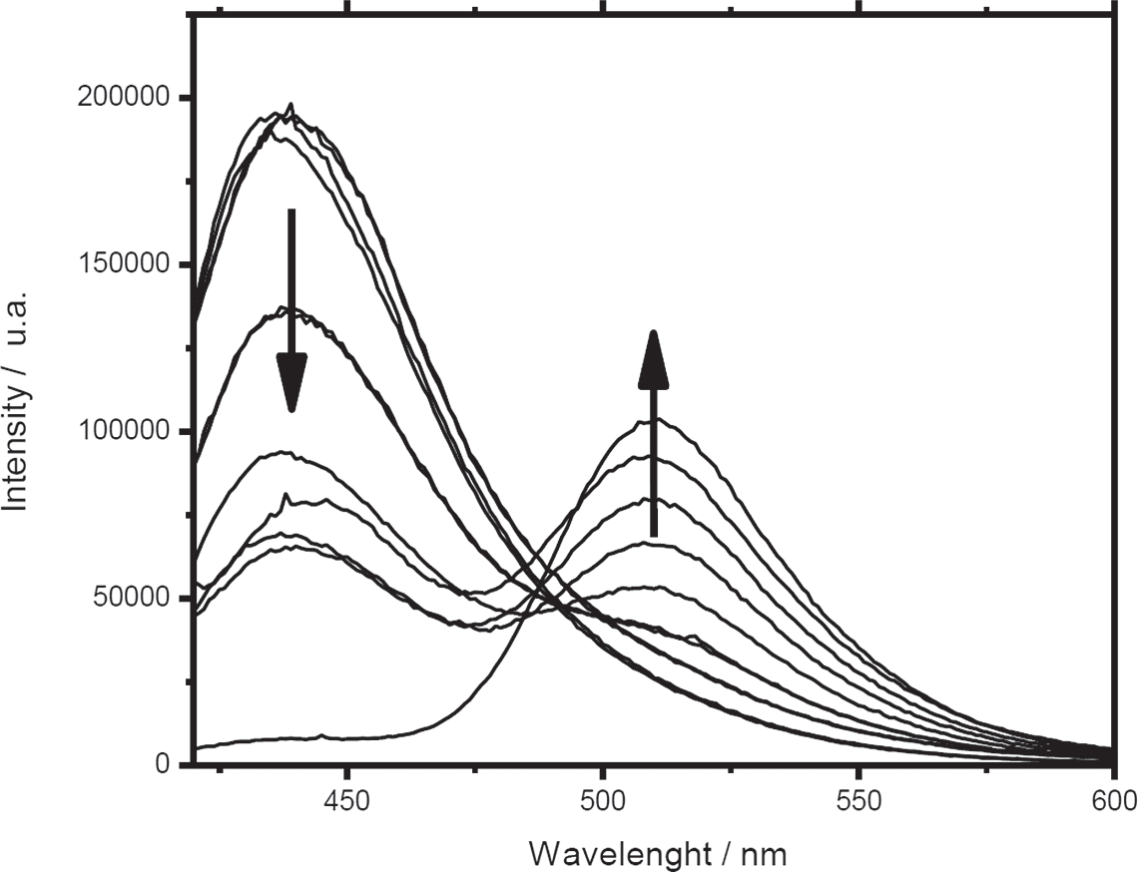
Emission spectra of Pyranine included in the pool of MPS/CHCl_3_ reversed micelles with increasing amounts of water. Arrows indicate the effect observed upon water increase.

To study the water structuration inside the aqueous pool of reversed micelles Pyranine was used. This molecule locates in the aqueous pool, and the analysis of the two emission bands of this molecule (440 nm and 510 nm) gives the information needed. Fig 5 shows the changes on the emission spectra of Pyranine inside reversed micelles of MPS as water inside the micelle increase. A decrease in the intensity at 440nm (PyOH*) with the simultaneous increase of the emission at 510nm (PyO^-^*) is observed as water content increased. This observation is a clear indication of the increase in ‘free-water’ (non-structured) inside the pool, able to shift the equilibrium to the PyO^-^*, the only excited emitting specie at high water content.

**Fig 6:**
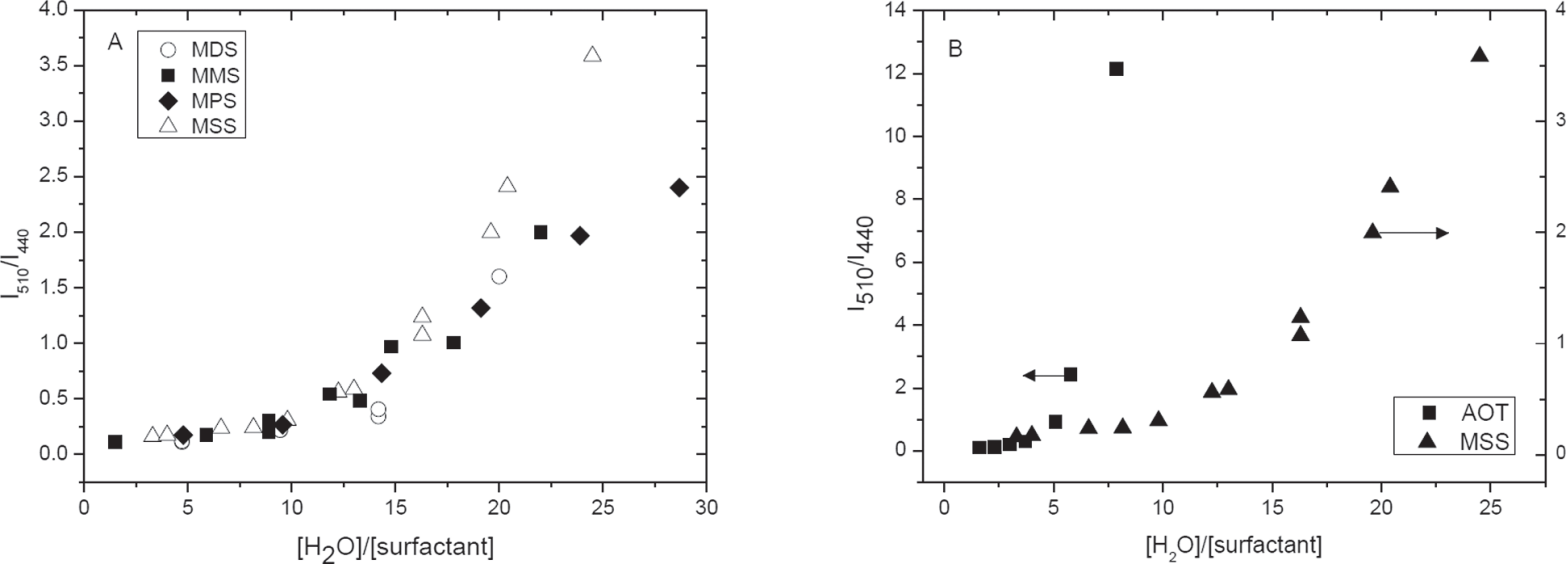
Intensities ratio for Pyranine emission as a function of water amount in the aqueous pool of reversed micelles ([H_2_O]/[surf]) formed with sucrose monoesters (A) and AOT (B), arrows indicate corresponding Y axis.

Fig 6 shows the emission ratio (I_510_/I_440_) for Pyranine as a function of the amount of water at the inside pools, represented by the ratio R ([H_2_O]/[surfactant]). Micro-emulsions for all sucrose monoesters (Fig 6A) showed a breaking point in the (I_510_/I_440_) ratio when R reaches values near 10. These results are an indication of free water detection: at R < 10, there is not free water to stablish the acid-base equilibria at excited state, therefore the presence of the basic excited specie (PyO^-^* with a maximum at 510nm) is negligible; At R > 10, the emission at 510 nm reports the shifting of the equilibrium toward the formation of PyOH* induced by the presence of free water molecules. Using the same methodology in reversed micelles of AOT (Fig 6B) similar results are observed with a breaking point at R = 5. These results are in agreement with reported data on water structuration for AOT reversed micelles using other methodologies,[2,41,42]

Our results indicate an important degree of water structuration in the aqueous pools of sucrose monoesters reversed micelles. We interpreted these results as follows: at low [H_2_O]/[surfactant] ratio, water molecules are forced to be located close to the hydrophilic heads, and their direct interaction with sucrose hydroxyl groups promotes a very high local viscosity. As a result water molecules are unavailable to participate in the acid-base equilibrium of Pyranine and the observed emission is dominated by the acidic specie (440nm, PyOH*). Zulauf et al. proposed that AOT reversed micelles below R = 10, behave like rigid macromolecules where water molecules are highly structured by hydrogen bonding and at the same time they are stabilized by the dipole moment of AOT head groups, and defining this behavior as Type I water.[41] When the amount of water in the aqueous pools increases, (values of R higher than the observed breaking point), the local viscosity decreases, the amount of ‘free’ water increases and the emission spectrum corresponds mainly to the emission assigned to the excited conjugated base of Pyranine, centered at 510nm. The behavior of this water molecules is almost identical to pure water and defined by Zalauf et al. as Type II water.[41]

The length of the alkyl chain does not show a noticeable effect on water structuration inside the aqueous pool of reversed sucrose ester micelles while all water molecules are interacting with sucrose moieties (as can be seen in Fig 5A, pyranine senses the same proportion of free water for all lengths of alkyl chain). When more water molecules start to become ‘free’ or available, the response of the probe is still the same for all sucrose esters. At higher values of R the response diverges depending on the alkyl chain.

When sucrose ester reversed micelles (Fig 5A) are compared with AOT ones (Fig 5B), two main differences are observed: (i) for sucrose ester there is a wider range of [H_2_O]/[surfactant] ratio values where water molecules behave as type I water (10 for sucrose esters and 5 for AOT). This observation indicates that the high number of hydroxyl groups on the sucrose moiety can organize effectively a larger number of water molecules as compared with the poly-oxyethylene chains of AOT and, (ii) for sucrose esters there is a smaller slope before the breakpoint, indicative of water molecules still organized in a solvation sphere around type I water.

We propose according this observation, that water structuration in sucrose ester reversed micelles modulates the excited state acid equilibrium of Pyranine: when water is completely structured around sucrose heads, the equilibrium is displaced towards the protonated form, and when free water is available, the equilibrium is favored towards the non-protonated specie.

#### 1.2 Rose Bengal (RB) Triplet state lifetime study

The measurements of triplet states lifetime give information about the properties of the microenvironment[43] around Rose Bengal probe. As mentioned in the methodology section, using Eq 2, we fitted the data from the phosphorescence decay of singlet oxygen from reversed micelles and we obtained the excited triplet lifetime of Rose Bengal and simultaneously the lifetime of singlet oxygen. In order to stablish the triplet lifetime of RB, the emission of singlet oxygen from deuterated water was fitted according Eq 2, yielding a growing component which corresponds to RB triplet lifetime, 3.4 μs, and a decay time of 66 μs, associated to the decay of O_2_(^1^Δ_g_) in this solvent. The fitting of decays obtained for singlet oxygen emission sensitized by RB in pure water, yielded a τ_1_ equal to 2.92 ± 0.08 μs, which corresponds to the sensitizer triplet lifetime, τ_T_, and τ_2_ with a value of 4.03 ± 0.35 μs, equal to the lifetime of singlet oxygen reported in water, τ_Δ_.[44]

**Fig 7:**
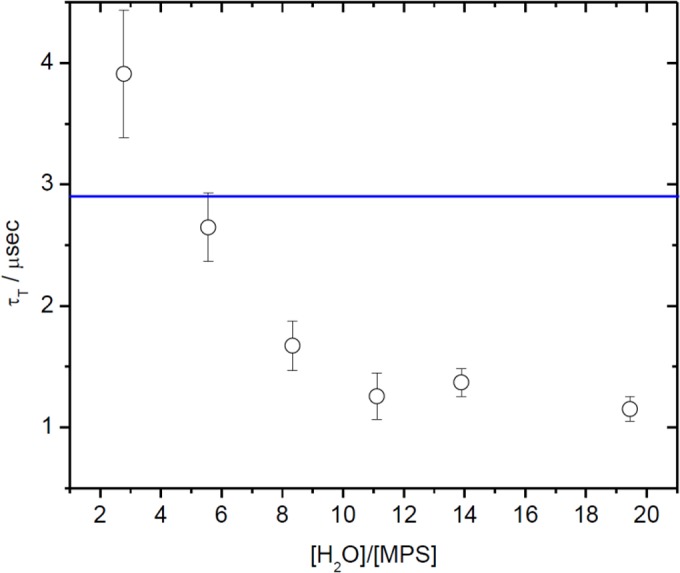
Bengal Rose triplet excited state lifetime in reversed micelles of MPS at different proportions of water. Line corresponds to the lifetime in pure water.

Fig 7 shows the changes in triplet state lifetime with increasing water content in the aqueous pool of MPS reversed micelles. The sensitizer triplet state lifetime showed a strong dependence with the amount of water present in the micro-heterogeneous system, a clear decrease of triplet state lifetime until plateau is reached around R = 10. For all the measurements performed, the lifetime assigned to singlet oxygen was 180.00 ± 13.00 μs, value in good agreement with the reported ones for chloroform in literature.[45,46]

Detailed observation of Fig 7 shows two interesting points: (i) we determined a triplet state lifetime value of 2.92 μs for RB in bulk water (blue line in Fig 7). In MPS reversed micelles, the lowest water content we measured was at R = 2 (this is the lower R value achievable considering the hydration water of sucrose esters and the aliquot of water where BR is dissolved) and we obtained τ_T_ of 4μs. This value is notoriously higher than the value determined in bulk water. We interpret this long-lived triplet state in terms of a highly structured media around the dye, which reduces molecular oxygen diffusion, with the concomitant reduction of quenching rate constant. (ii) At R values around 6 the triplet state lifetimes show a similar value (3 μs) to the one determined in bulk water, an expected result however (iii) the triplet state lifetime continue dropping as the R value increase (water content inside the micelle increase) until reaching a plateau around 1.5 μs. The lifetime of a triplet state in the presence of oxygen depends on the viscosity of the media (which affects diffusion) and on the oxygen concentration. If we consider similar fluidity of bulk water and water on the center of the pool, we can explain the higher efficiency of quenching of the triplet states as a consequence of a higher oxygen concentration inside the reversed micelles.

### 2. Study of the effect of the presence of sucrose monoesters on the palisade of Triton X-100 micelles

#### 2.1 Steady state measurements

Changes in the microstructure of micelles are often analyzed by exploring two micro-environmental properties, namely micropolarity and microviscosity. We used Pyrene to examine modifications in the micropolarity of Triton X-100 micelles. This fluorescent probe solubilized in the micelar palisade layer, close to the micelar surface and the I/III ratio (described in methodology) is used to report alterations in the degree of solvation of the micelles.[47] To examine changes in micellar viscosity, Laurdan, was used. This fluorescent probe, used to measure dipolar relaxation,[36,48] is expected to be anchored in the hydrophobic core of Triton X-100 micelles[36] and the GP value (methodology) is related with microviscosity.[49]

Table 1 shows the Pyrene I/III ratio and Laurdan GP values obtained for pure and mixed micelles of SEs and Triton X-100. For pure Triton X-100 micelles, the 1.3925 value for the I/III ratio indicate a polarity similar to methyl alcohol,[35] and it is consistent with data reported in literature.[50] Laurdan GP value of -0.1250 for pure Triton X-100 micelles indicates a system with relatively low fluidity, and will be the reference value for the mixed micelles. The values for I/III ratio on pure SEs micelles are lower than the one obtained for Triton X-100 (1.1390, 1.1087 and 1.0130 for MCS, MLS and MPS respectively) indicating a less polar environment on the probe location for the three SEs. Laurdan GP values measured for pure MLS (-0.3400) and MPS (-0.3600) micelles are significantly lower than those for Triton X100, indicating a more fluid interface for pure SEs micelles and therefore better water accessibility. For the MCS micelles, the Laurdan GP value (-0.0804) indicates a more compact structure. We interpreted this data in good correlation with different size of the micelles and a different packing of head groups. The MLS and MPS having longer hydrophobic tail (11 and 15 methylene units respectively) would form larger micelles than MCS (9 methylene units).

**Table 1:** Effect of presence of increasing amounts of the different sucrose monoesters in micropolarity and microviscosity Triton X100 micelles.

	MCS		MLS		MPS	
χ_(X-100)_	I/III	GP	I/III	GP	I/III	GP
1.00	1.3925	-0.1250	1.3925	-0.1250	1.3925	-0.1250
0.75	1.1360	-0.0982	1.3599	-0.1058	1.1740	-0.1376
0.50	1.1300	-0.1439	1.3020	-0.1970	1.1370	-0.0924
0.25	1.1280	-0.0749	1.2004	-0.1840	1.0930	-0.1417
0.00	1.1390	-0.0804	1.1087	-0.3400	1.0130	-0.3600

**Fig 8:**
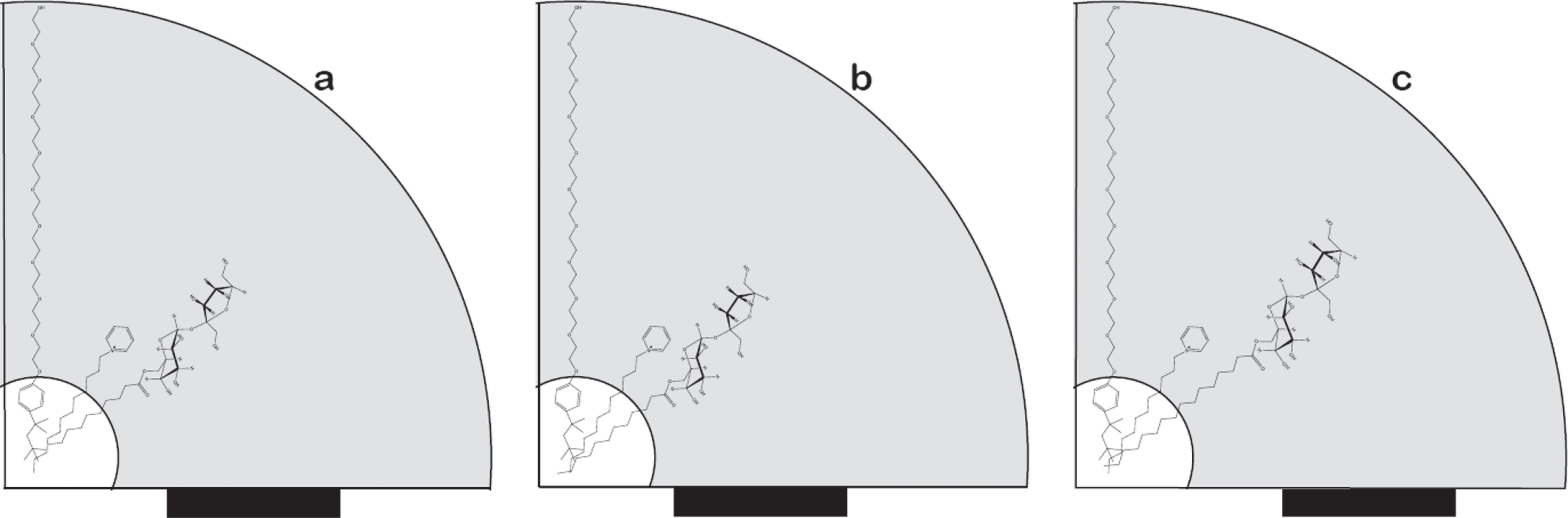
Rough diagrammatic representation of Triton X-100 micelles, considering chains fully extended. The inner core (white) corresponds to the hydrophobic nucleus, and the grey region corresponds to the palisade. Black cursor below indicates sucrose moiety position in the palisade for MCS (a), MLS (b) and MPS (c). Also DPC localization is indicated.

The presence of increasing amounts of MLS on Triton X-100 micelles induces a gradual decrease in the sensed micropolarity (I/III ratio), while the incorporation of MCS or MPS produce abrupt changes. These results indicate alteration of the Triton X-100 palisade with SEs incorporation dependent on the SEs size and the hydrophilic lipophilic balance (HLB) of the molecules: MLS molecule, with 11 methylene units is anchored to hydrophobic core, with sucrose moiety Triton X-100 micelle, therefor the observed changes will be gradual. MPS with 15 methylene units will be forced to have the sucrose head into the palisade and the MCS molecule (with 9 methylene groups) would have similar location due to its lower hydrophilic/lipophilic balance. (see Fig 8)

#### 2.2 Time resolved measurements

The fluorescence decay of Pyrene strongly depends on the microenvironment where it is located. We studied the fluorescent decay of this dye when is solubilized on pure SEs, pure Triton X-100 and mixed micelles. We evaluated the decay parameters for Pyrene emission using dodecyl pyridinium chloride (DPC) as quencher. On Triton X-100 micelles, DPC has been reported to migrate between micelles and its exchange rate would be influenced by the palisade. The use of DPC as quencher for Pyrene is appropriate for the experiments we are performing in this study however, it is reported to be inappropriate for aggregation number determination on Triton X-100 micelles.[51]

Decay of Pyrene fluorescence in the presence of increasing amounts DPC as quencher, were acquired for different mixed micelles. Data were recorded for several occupancy numbers of DPC (the highest occupancy numbers employed in our experiences were 1.5. Data were fitted using the model described for mobile quencher-immobile probe (Ec. 4, where the probe remains in a single micelle but the quenchers are able to exchange between micelles during probe lifetime). Pyrene lifetime, exchange rate constants and quenching rate constants were defined as global parameters for the complete set of decays. Table 2, summarizes the results obtained using this time-resolved fluorescence procedure to fit response in micelles of triton X-100 mixed with the three different sucrose esters in different proportions.

**Table 2:** Effect of sucrose esters in the quenching process of Pyrene by dodecylpyridinium chloride (DPC) in pure and mixed Triton X-100 micelles.

Sucrose esters	χ_Triton X-100_	k_0_/10^6^seg^-1^	k_-_/10^6^seg^-1^	k_q_/10^6^seg^-1^
	1	4.26	3.32	6.42
MCS	0.8	3.92	9.55	7.59
	0.6	3.97	7.77	2.32
	0.5	3.95	7.43	4.29
	0	3,57	-	1.11
MLS	0.8	4.51	2.60	6.37
	0.6	4.43	2.35	6.34
	0.5	4.45	1.91	6.29
	0	4.09	0.44	7.10
MPS	0.8	4.14	3.94	7.75
	0.6	4.36	7.67	12.8
	0.5	4.22	9.41	15.3
	0	4.05	-	3.71

For pure MLS micelles the Pyrene decay data fitted to Eq 4 yields a quencher exit rate (k_-_ = 4.4 x 10^5^ seg^-1^) ten-fold smaller than the exchange rate constant observed for Triton X-100 micelles (3.32 x 10^6^ seg^-1^). For pure micelles of MCS and MPS, data did not fit to Eq 4 but the model described by Eq 3 for immobile quencher-immobile probe results to be appropriate. Thus, for pure micelles of MCS and MPS, DPC is not able to exchange between micelles during Pyrene lifetime.

Pyrene lifetime shows no dependence on the nature of the surfactant used, being the k_o_ values of similar magnitude for Triton X-1000, MLCS, MLS and MPS (Table 2). This behavior indicates that Pyrene senses a similar environment in all cases, as shown also by the Pyrene I/III ratio, therefore, we can conclude that Pyrene is localized in regions of similar properties for all cases.

The quenching rate constant, gives information on the easiness for the quencher to reach the probe. This constant is similar for Triton X-100 (6.42 x 10^6^ seg^-1^) and MLS (7.1 x 10^6^ seg^-1^) micelles, again showing some sort of match, but the quencher diffusion towards Pyrene is more difficult in MPS micelles where k_q_ equals 3.71 x 10^6^ s^-1^ and remarkably in MCS micelles, where quenching rate constant is only 1.1 x 10^6^ s^-1^. These results are also congruent with the ability of quencher to release micelles, determined for each system.

By employing data reported in a previous paper,[52] in a coarse calculation (involving hydrodynamic radius and aggregation numbers) superficial area of each sucrose moiety is near 1.65nm^2^ for both MCS and MPS micelles while for MLS this value is only 1.30nm^2^, these values indicate a different degree of packing of sucrose molecules in the micellar interface.

In the mixed micelles, the incorporation of SEs, induces structural changes in the palisade dependent on the amount of sucrose present as shown by the values of k_-_ and k_q_ (Table 2). On the other side, Pyrene lifetime does not depend on the amount of sucrose present in the palisade, suggesting again, that either the probe is located in an area where it is not affected by the presence of sucrose or the probe moves and relocates according to the amount of sucrose present, in which case the changes promoted on the palisade would not be detected.

The changes on the quencher exchange and quenching rate constants promoted by the presence sucrose ester in Triton X-100 micelles is different for each SE. The presence of both, MCS and MPS facilitates the exchange of the quencher (reflected in greater values of k_-_), while MLS fairly blocks the quencher exit. For mixed micelles with the shortest and the longest alkyl chains (MCS and MPS) the presence of sucrose moieties induces larger disturbance in the micelle core, facilitating the exit of the quencher from the micelles.

The values obtained for fluorescence quenching rate constant, related primarily with micelle viscosity, show a different behavior for each sucrose ester in MCS/Triton X-100 systems the effect of increasing the SE concentration is to decrease the values of k_q_. We interpreted this results as a hindering effect of the sucrose moiety on the Pyrene-quencher interaction. Following the same reasoning, for MLS/-Triton X100 mixed there is no noticeable effect (rate constant remains almost constant) and finally for MPS-X100 mixed systems the increasing presence of the ester facilitates notoriously the quenching process.

Our results, indicate that the presence of sucrose moieties induces changes in the structuration of the palisade by increasing the number of hydrogen bonds with water molecules. Under this scenario, the length of methylene chain attached to the sucrose would play a role in the blocking ability of this barrier. Fig 8 shows a diagram with the main conclusions from this results: for SEs:Triton X-100 mixed micelles with low SEs proportion, sucrose moieties are located at different depths inside the poly-oxyethylene palisade depending on the size of the methylene tail and the hydrophilic lipophilic balance (HLB) of the molecules. MCS molecule (7a), with 9 methylene groups, would locate the sucrose moiety closer to the center of the Triton X100 palisade with almost half of the tail in the hydrophobic core. The MLS (Fig 7B), with 11 methylene units, would locate their sucrose moiety near the hydrophobic core and would have almost all the hydrophobic tail anchored on the hydrophobic core (Fig 7B). MPS, with 15 methylene groups would be forced to have the sucrose moiety closer to the micelle surface in order to accommodate the hydrophobic tail. The quenching of Pyrene by DPC would be affected by the SE present in the micelle. In MCS:Triton X-100 micelles, DPC would be located near the carbonyl group of the ester, and Pyrene in the external region of palisade. Under these conditions, DPC can exit the micelle since is loosely anchored and the interaction of Pyrene with DPC is blocked by the sucrose moiety. For MLS:Triton X-100 micelles we propose DPC would interact directly with sucrose, limiting its exchange rate, and Pyrene can reach easily the quencher. Finally for MPS:Triton X-100 micelles with DPC located below the sucrose moieties, we propose that Pyrene is located in same region of the palisade (moving away from the upper palisade, in more polar environment consequence of sucrose interference), in this condition the quencher is able to exit from micelle, and encounters with Pyrene are facilitated.

This description also accounts for the differences observed on the capacity of the quencher to reach the probe (k_q_) and the exit rate of the quencher from the micelles (k_-_). According Gehlen et al., in the absence of electrostatic interactions, the exit of the quencher from the micelle would depend only on the hydrophobic interactions of the quencher and the micelar host structure.[40] Our results show that besides hydrophobic interactions, in mixed micelles (SE and Triton X-100) the mismatch between the hydrophobic regions forming the apolar core would have a remarkable effect on the capacity of quencher to exchange between micelles.

In summary, in this study we expand and clarify several aspects on the effect of sucrose moieties presence at the interface of direct and reverse micelles of sucrose monoesters and studied the alterations observed in the palisade in mixtures of Triton-X100 with sucrose esters (combination of surfactants not previously reported).

For SEs reversed micelles, the structuration of water inside aqueous pool in the interface can be easily detected. Pyranine experiments show SEs are able to interact with more water molecules than AOT. This water structuration, as can be seen in RB experiments, promotes a higher viscosity which affects excited state reactions.

In pure direct SE micelles, quenching of Pyrene emission is not only affected by the presence of sucrose moieties at the interface, but also by the length of hydrocarbon chain. DPC quenching rate constant has comparable values for MLS and Triton micelles, indicating similar viscosity for both systems. In the case of MCS and MPS micelles a higher viscosity is presumed because quencher movement is restricted when compared with pure Triton micelles. The barrier formed by sucrose moieties on the surface of pure micelles is more effective than the polyoxyethylene palisade of Triton X-100 limiting quencher exchange between micelles. Our results indicate that Triton X-100 palisade, representing a steric hindrance, can be overpassed by species like DPC, while sucrose moieties able to form a tighter blockade which is an impediment for DPC exit.

For mixed direct SE/Triton X-100 micelles, the expected improvement in the blocking effect of a palisade is not observed for all SEs. The presence of sucrose moieties in the palisade has different effects depending on the number of methylene units of surfactant hydrophobic tail. The disturbing effect on the micelle core, consequence of the difference in size of both hydrophobic tails (Triton X-100 and SE), plays an important role, and the blocking effect can be overcame when the hydrophobic tails of SEs are too short or too long compared with Triton alkyl chain. Only for MLS (eleven methylene units) the increase of its proportion yields an interface less susceptible to allow quencher migration.
